# Cyclic Vomiting Syndrome (CVS): is there a difference based on onset of symptoms - pediatric versus adult?

**DOI:** 10.1186/1471-230X-12-52

**Published:** 2012-05-28

**Authors:** Nilay Kumar, Qumseya Bashar, Naveen Reddy, Jyotirmoy Sengupta, Ashwin Ananthakrishnan, Abigail Schroeder, Walter J Hogan, Thangam Venkatesan

**Affiliations:** 1 Department of Gastroenterology and Hepatology, Medical College of Wisconsin, 4th Floor, Froedtert East Building, 9200 W Wisconsin Avenue, Milwaukee, WI, 53226, USA; 2 Mayo Clinic Florida, Florida, USA; 3 Wayne State University, Detroit, USA; 4 Massachusetts General Hospital, Massachusetts, USA

**Keywords:** Motility, Cyclic vomiting, Nausea, Abdominal pain

## Abstract

**Background:**

Cyclic Vomiting Syndrome (CVS) is a well-recognized functional gastrointestinal disorder in children but its presentation is poorly understood in adults. Genetic differences in pediatric-onset (presentation before age 18) and adult-onset CVS have been reported recently but their clinical features and possible differences in response to therapy have not been well studied.

**Methods:**

This was a retrospective review of 101 CVS patients seen at the Medical College of Wisconsin between 2006 and 2008. Rome III criteria were utilized to make the diagnosis of CVS.

**Results:**

Our study population comprised of 29(29%) pediatric-onset and 72 (71%) adult-onset CVS patients. Pediatric-onset CVS patients were more likely to be female (86% vs. 57%, p = 0.005) and had a higher prevalence of CVS plus (CVS + neurocognitive disorders) as compared to adult-onset CVS patients (14% vs. 3%, p = 0.05). There was a longer delay in diagnosis (10 ± 7 years) in the pediatric-onset group when compared to (5 ± 7 years) adult-onset CVS group (p = 0.001). Chronic opiate use was less frequent in the pediatric-onset group compared to adult-onset patients (0% vs. 23%, p = 0.004). Aside from these differences, the two groups were similar with regards to their clinical features and the time of onset of symptoms did not predict response to standard treatment. The majority of patients (86%) responded to treatment with tricyclic antidepressants, anticonvulsants (topiramate), coenzyme Q-10, and L-carnitine. Non-response to therapy was associated with coalescence of symptoms, chronic opiate use and more severe disease as characterized by longer episodes, greater number of emergency department visits in the year prior to presentation, presence of disability and non-compliance on univariate analysis. On multivariate analysis, only compliance to therapy was associated with a response. (88% vs. 38%, Odds Ratio, OR 9.6; 95% Confidence Interval [CI], 1.18-77.05).

**Conclusion:**

Despite reported genetic differences, the clinical features and response to standard therapy in pediatric- and adult-onset CVS were mostly similar. Most patients (86%) responded to therapy and compliance was the only factor associated with a response.

## Background

Cyclic vomiting syndrome (CVS) is a chronic gastrointestinal disorder that was initially described in children but is now being recognized with increasing frequency in adults [1-3]. Rome III criteria for CVS in adults includes stereotypical episodes of vomiting regarding onset (acute) and duration (less than 1 week); 3 or more discrete episodes in the prior year and absence of nausea and vomiting between the episodes [4]. Adult CVS can range from mild disease with infrequent episodes to severe life-disabling disease requiring multiple emergency department (ED) visits and frequent hospitalizations [1,5,6]. Since CVS is often unrecognized, these patients often undergo a battery of unnecessary diagnostic and therapeutic procedures without any clinical benefit [1,7]. Many patients, if not treated appropriately, develop coalescence of symptoms with interepisodic nausea and loss of the periodicity of episodes. Several CVS patients also undergo unnecessary surgical procedures such as cholecystectomy for symptoms caused by CVS [7].

CVS may be categorized as pediatric- and adult-onset CVS based on the age of onset of symptoms. Pediatric-onset CVS patients develop symptoms prior to the age of 18 while adult-onset CVS patients develop symptoms ≥ 18 years [1,8]. Genetic differences have been identified in these two groups with mitochondrial DNA (mtDNA) mutations, 16519 T and 3010A being associated with pediatric-onset CVS [2,9-11].

Strong maternal inheritance of multiple disease manifestations and abnormal urine organic acids has been demonstrated in children with CVS, suggesting the presence of predisposing mtDNA sequence polymorphisms [12,13]. It has also been reported that pediatric-onset CVS patients have a higher proportion of maternal inheritance of functional symptomatology as ascertained by quantitative pedigree analysis [14].There are presently no data that have elucidate the clinical differences and response to treatment pertaining to these groups . Based on our experience with a large cohort of CVS patients seen at the adult CVS clinic in Milwaukee, we hypothesized that despite these genetic differences, both groups of patients had similar clinical characteristics and response to standard medications used in the treatment of CVS. For treatment of these patients we also utilized the standard medical regimen shown to be effective in existing literature [15].

## Aims

The primary aims of our study were 1) To define the demographics and clinical characteristics and assess response to therapy in pediatric-onset and adult-onset CVS patients. 2) To identify predictors of response to treatment in both groups of patients.

## Results

### Study population

Of 101 patients, there were 66 (65%) females. There were 79 (79%) whites, 17(17%) blacks, 3 (3%) of hispanic origin and 1 (1%) of other origin. Six patients met the diagnostic criteria for CVS plus whereas 10 patients met criteria for catamenial CVS (episodes triggered by menstrual periods) [1]. CVS plus is defined as CVS in association with neurocognitive disorders [16]. The mean age at presentation was 27 ± 12.3 years. The median duration of an episode was 3 days (range 1-18 days) with 81(81%) patients reporting a prodromal phase. At the initial clinic visit, 39 (39%) patients had coalescence of symptoms with daily or near-daily symptoms but had typical episodes at the outset. Patients with coalescent CVS had a longer delay in diagnosis as compared to typical CVS patients (91 ± 86 vs. 75 ± 85 months) though this was not statistically significant. Of all patients, 63(62%) had abdominal pain as a prominent symptom during episodes along with nausea and vomiting. The location of the abdominal pain was variable with 40 patients (63%) having generalized abdominal pain, 14 patients (22%) reporting upper abdominal pain and 9 patients (15%) with pain in the lower abdomen. Almost all patients (90%) had nausea as a part of their symptom complex and 76 patients (76%) stated that the onset of episodes was in the morning. Data on hot and cold showers was available in 73 patients; 38 (52%) patients had symptom relief with hot showers and 1 patient had relief with a cold shower. Hot showers were reported in 25/35 (71%) CVS patients using marijuana when compared to non users 19/56 (34%, p = 0.01).

Triggers for CVS episodes were reported in 87(87%) of patients with the most common triggers being negative stress (unpleasant or sad events) in 67(67%) and positive stress (happy or exciting events) in 59(59%). The recovery phase was very variable and ranged from 10 minutes – 7 days. Many patients had concomitant co-morbid conditions such as anxiety (47%), depression (49%) and dysautonomia (64%). Gastric emptying studies were available for review in 40 patients; 10 patients (25%) had gastroparesis whereas 4 patients (10%) had rapid gastric emptying and 26 (65%) had normal emptying.

Thirty of seventy (43%) patients had a personal history of migraine and 41/64(64%) had a family history of migraine. Most of the patients in our cohort had undergone numerous diagnostic and therapeutic procedures; 28/92 (30%) had undergone a cholecystectomy for symptoms of CVS without any therapeutic benefit. Eighteen of 75 patients (24%) reported some form of disability from CVS. Sixteen (24%) of 66 patients had a delay in higher education and 20 of 70 patients (29%) reported job loss related to CVS episodes.

### Pediatric- vs. adult-onset CVS

In our cohort of 101 adults with CVS, twenty nine patients (29 %) had pediatric-onset CVS as compared to 72 (71 %) with adult-onset CVS. The demographics, clinical characteristics, associated co-morbid conditions and response to treatment are depicted in table 1. Patients were predominantly white in both groups but more patients were female in those with pediatric-onset CVS 25 (86 %) than adult-onset CVS 41(57 %), (p = 0.005). Both subgroups had similar disease characteristics with no significant differences noted in trigger factors, co-morbid conditions, rate of surgery for CVS symptoms, or personal/family history of migraine. Coalescence of symptoms was noted in both pediatric- and adult-onset CVS. There were no significant differences in response to treatment in both groups of patients (p = 0.27).

**Table 1:** Demographic and disease characteristics of patients with CVS based on disease onset

**Variables**	**Pediatric Onset (n-29),**	**Adult Onset (n-72)**	**p-value**
Mean age at onset of symptoms(years)	13.4±12.5	32.3±12.3	***0.0001***
Female sex	25 (86%)	41 (57%)	***0.005***
**Race (%)**			
White	25 (86%)	54 (75%)	0.29
Black	3 (10%)	14 (20%)	0.38
Hispanic	1 (4%)	2 (3%)	1
Other	0	1 (1%)	
**Type of CVS**			
CVS plus (%)	4 (14%)	2 (3%)	***0.05***
Catamenial CVS	5 (17%)	5 (7%)	0.15
Sato’s CVS	2 (7%)	4 (6%)	1
Delay in diagnosis (months)	124.75±85.86	63.53±85.23	***0.001***
Coalescence	14 (48%)	25 (36%)	0.25
**Associated Symptoms**	**Available n = 27**	**Available n = 67**	
Abdominal Pain	16 (59%)	47 (70%)	0.34
**Co-morbid disorders**	**Available n = 28**	**Available n = 68**	
IBS	9 (32%)	18 (27%)	0.62
Anxiety	13 (46%)	32 (47%)	0.19
Depression	9 (32%)	33 (49%)	0.18
Bipolar Disorder	1 (4%)	1 (2%)	0.5
Migraine	16 (55%)	30 (43%)	0.28
Cholecystectomy	9 (32%)	19 (30%)	0.81
**Family History (%)**	**Available n = 25**	**Available n = 64**	
Family history of migraine	16 (64%)	41 (64%)	1
**Drug use %**	**Available n = 27**	**Available n = 65**	
Marijuana	8 (30%)	27 (42%)	0.35
Alcohol	7 (26%)	10 (15%)	0.25
Tobacco	3 (12%)	30 (46%)	***0.001***
Narcotics	0	15 (23%)	***0.004***
Response to therapy	19/20 (95%)	47/56 (84%)	0.27

Pediatric-onset CVS patients differed from adult-onset CVS in having a higher prevalence of CVS-plus. (14% vs. 3%, p = 0.05). There was a marked delay in diagnosis in both groups but those with pediatric-onset CVS has a much longer mean delay in diagnosis in comparison to adult- onset CVS (10 ± 7 vs. 5 ± 7 years, p = 0.001). Adult-onset CVS patients had a significantly higher incidence of tobacco and narcotic use of 46% and 23% when compared to 12% and 0% in the pediatric-onset group (p = 0.001 and 0.004 respectively).

### Treatment profile of patients with CVS and variables associated with response to treatment

**Figure 1:**
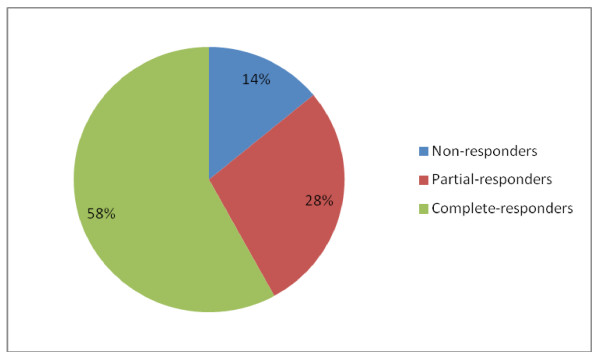
Response profile of CVS patients.

**Figure 2:**
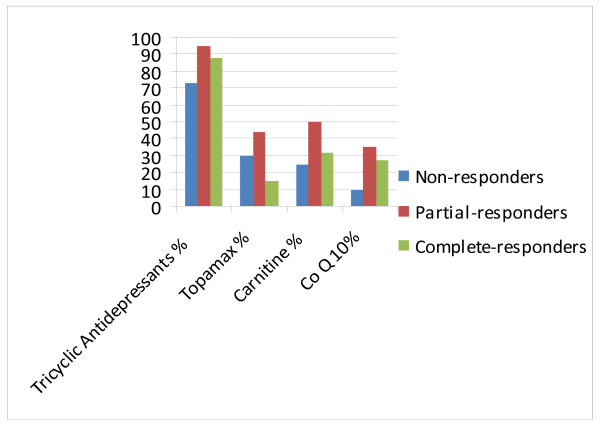
Medications used in complete, partial and non-responders did not reveal any significant differences.

Most patients 70/92 (76%) were on tricyclic antidepressants (TCA) such as amitriptyline or nortriptyline, 18 /92 patients (20%) on topiramate and 27/92 (30%) on coenzyme Q-10, L-carnitine or riboflavin. Of the 77 patients with adequate data on response to triptans as abortive therapy, 64 (83%) patients were able to abort episodes. Of the 70 patients on tricyclic antidepressants, 18 patients (26%) had to stop the medication due to drug intolerance; the most common adverse effects included bad dreams, behavioral changes and increased somnolence. The mean dose of TCA used in patients who tolerated therapy was 83.3 ± 36.6 mg. Adequate follow up data was available in 76/101(75%) patients (75%) who were initiated on medical therapy and mean duration of follow-up was 11.2 ± 6.2 months. Response to treatment in these patients is depicted in figure 1. The majority of patients responded to treatment with 44 (58%) patients having a complete response, 21(28%) a partial response and 11(14%) patients with no response to therapy. There were no significant differences in the type of medications or dosages used in complete, partial and non-responders. (Figure 2)

The demographics and clinical characteristics of responders (both complete and partial) and non-responders are depicted in table 2. On univariate analysis, non-responders were more likely to have a pattern of coalescence (82% vs. 34% p = 0.005). They also had had longer episodes lasting 6.5 days vs. 3.5 days and greater numbers of ED visits (15 vs. 3) in the year prior to evaluation compared to responders. The incidence of chronic opiate use was significantly higher amongst non-responders than responders (46% vs. 13%, p = 0.02). A significantly higher rate of disability amongst non-responders was also noted (57 % vs. 20 %, p = 0.05). Non-responders were less likely to be compliant to treatment (38 % vs. 88 %, p = 0.004). On multivariate analysis only compliance was found to be statistically significant in predicting response to treatment (p = 0.03, OR 9.6, 95 % CI 1.18-77.05).

**Table 2:** Univariate analysis of responders and nonresponders to treatment

**Variables**	**Responders [Partial n-21; Complete n-44]**	**Non-Responders (n-11)**	**p-value**
Pediatric onset	31	18	0.49
Gender	63	64	1
Females %			
Race			
White %	82	82	1
Black %	14	18	0.66
Coalescence %	34	82	***0.005***
Narcotics %	13	45	***0.02***
Compliance %	88	38	***0.004***
Loss of job %	31	57	0.21
Disability %	20	57	***0.05***
Surgery for CVS %	29	30	1
No. of episodes/yr	31.3±50	46.6±52.8	0.4
Duration of episodes (days)	3.5±3.5	6.5±3.5	***0.008***
Warning phase (Minutes)	159.2±365	47.1±378.1	0.45
Recovery Phase (days)	1.7±1.9	2.2±1.9	0.43
No. of emesis/hr	12.2±17.1	19.1±17.4	0.31
ED visits last year	3.4±9	15.8±9.2	***0.0002***
Number of hospitalizations	11.5±31.8	31±32	0.08
Average duration of hospitalizations	5.1±8.8	3.3±8.8	0.57

## Discussion

The salient findings of our study are that both adult- and pediatric-onset CVS patients have a similar demographic profile and disease characteristics except that pediatric-onset patients were more likely to be female and had a higher prevalence of CVS-plus with concomitant neurological disorders. This finding may be explained by the genetic differences that have been elucidated in previous studies [2,10,11]. The pediatric-onset group also had a lower incidence of tobacco and opiate use and a significantly longer delay in diagnosis of CVS. This longer delay in diagnosis is an unexpected finding given that CVS is fairly well-recognized condition in children. It should be noted that though our center attracts both children and adults with CVS, the majority of the patients with pediatric-onset CVS were not transitioned from the Children's Hospital of Wisconsin, which is also a tertiary center for pediatric CVS. Dysautonomia was diagnosed in 64 % of our CVS patients which we recently reported to be a significant problem in this population [17]. Similar to reported literature, CVS patients in both groups had a strong personal or family history of migraine [18,19]. Co-morbid conditions including anxiety, depression and irritable bowel syndrome were seen in both groups and it is unclear if this is related to the lack of receiving a credible diagnosis and the inordinate delay in treatment that these patients experience. In addition, 30 % of patients underwent invasive surgery for CVS symptoms without any benefit. Long delays in diagnosis along with such unnecessary and unhelpful interventions are unacceptable both from a patient standpoint and from an economic perspective.

The majority of our patients (86 %) had either a complete or partial response to prophylactic medications with TCA’s, topiramate and mitochondrial therapy such as carnitine, coenzyme Q-10 and ribofavin. The dose of carnitine used was 1 gram twice daily, co-enzyme Q-10 was 200 mg twice daily and riboflavin 100 mg once daily. On univariate analysis, non-response to therapy was associated with coalescence of symptoms, chronic opiate use and more severe disease as characterized by longer episodes, greater number of ED visits in the year prior to presentation, presence of disability and non-compliance. Compliance was the only significant variable on multivariate analysis that predicted response to therapy. Patients were deemed noncompliant if they continued to use marijuana and it is the practice of the author to advise complete abstinence from marijuana in all patients. We did not routinely check for cessation of marijuana in our patients with toxicology screens. There was no difference in response to therapy between pediatric-onset and adult-onset CVS patients despite recent reports of genetic differences. This may imply that there are other factors involved in the pathogenesis of this disorder aside from mitochondrial abnormalities in adults.

The few patients who were administered TCA’s prior to being seen at our clinic were on a low daily dose of 25 mg that is employed in other functional gastrointestinal disorders. Dose escalation to 1 mg/kg as used in children produced the desired therapeutic effect. However, there was a high incidence of side effects associated with TCA therapy resulting in discontinuation in 26% of patients which limited its use [20].

Marijuana use was seen in more than a third of patients with CVS and patients frequently reported using this for alleviation of nausea and as an appetite stimulant. There did not appear to be any clear cause-and-effect or temporal relationship between the use of marijuana and onset of symptoms based on clinical history and these patients were not thought to have symptoms caused by the use of marijuana. Marijuana use has been thought to result in cannabinoid hyperemesis [21-23]. Though the patients reported in the literature have not had adequate follow up; this entity as a separate disorder is subject to controversy. This raises questions about whether chronic marijuana use down regulates CB1 receptors and paradoxically causes more nausea and vomiting which will need to be evaluated in the future studies.

We acknowledge that there are several limitations to this study as this was a retrospective analysis though the data was also obtained prospectively with a standardized questionnaire used in clinic. The response to medications has been arbitrary in CVS thus far and there has been no consensus or accepted definitions in adult patients for measures of response to treatment in CVS. ED utilization has been recently studied in CVS patients; we used overall improvement of symptom frequency and severity to assess response to treatment [24]. We were unable to report on exact numbers of ED visits prior to and after treatment as many patients visited other ED’s for CVS episodes. In addition while other authors have measured ED visits and hospitalizations as outcomes of therapy, some patients with CVS have home intravenous fluid therapy or are seen in infusion clinics as in our practice to help control their symptoms and avoid long waits in the ED and prevent hospitalizations. Our study is one of the largest studies characterizing adult-onset and pediatric-onset CVS patients. This study should pave the way for future prospective evaluation of treatment in patients with CVS and standardized measures of disease outcomes.

## Conclusion

In conclusion, adult-onset CVS patients have similar symptoms to those with pediatric- onset CVS and have similar rates of response to therapy. CVS patients appear to respond favorably to higher doses of TCA’s of 1 mg/kg in comparison to the lower doses that are used in other functional gastrointestinal disorders. CVS is associated with an enormous psychosocial and economic burden and patients must be diagnosed early and treated with appropriate medications. Combination of medical and behavioral therapy based on a bio-psycho-social model is key to successfully treating patients.

## Methods

### Study population

A retrospective review of 101 adults with CVS seen at the Medical College of Wisconsin between September 2006 and October 2008 was performed. The adult CVS clinic at the Medical College of Wisconsin (MCW) serves as a tertiary referral center for patients with CVS. Patients were identified based on the Rome III criteria for CVS and comprised of patients from 15 states in the USA and Canada. Data was collected at the time of initial patient visit and subsequent follow up visits. All patients had stereotypical episodes at onset but some patients developed coalescence of symptoms later when they developed significant interepisodic nausea and dyspepsia and lost the typical periodicity seen during the initial phase of their illness. Patients with coalescence of symptoms were included only if they had episodic symptoms at the outset.

### Data collection

Demographic characteristics including age, gender, race, geographic location, personal and family history of all patients were collected at the time of initial evaluation with a standard questionnaire that was completed by patients prior to their appointment. This was then reviewed at the time of the initial clinic visit and information was confirmed. Data was collected on disease characteristics such as duration and severity of disease, age of onset, delay in diagnosis, specific triggers, associated co-morbid conditions, history of marijuana use and number of emergency department (ED) visits and hospitalizations. Medications used as prophylactic therapy in CVS including tricyclic antidepressants such as amitriptyline and nortriptyline, anti-epileptics such as topiramate and mitochondrial supplements such as coenzyme Q-10, L-carnitine, and riboflavin were recorded. Adverse effects of medications, response to treatment, disability and job loss due to CVS were ascertained. Data was collected on a Microsoft Excel spreadsheet; most of the variables were in a categorical format with some data such as number of ED visits and hospitalizations recorded as continuous variables. Data was collected as per the historical recollection of the patient with supplementation from previous medical records. The patients were divided based on time of onset of symptoms (pediatric-onset and adult-onset) and response to therapy [1]. This study was approved by Institutional Review Board at the Medical College of Wisconsin.

All patients seen in our clinic were asked standard questions about the frequency, duration and severity of episodes. Complete response to therapy was defined as ≥ 80 % amelioration in symptom duration, frequency and severity and partial response was at least a 50-80 % reduction. Non-responders had either no change in their disease status or < 50 % reduction in symptom duration, frequency and severity. Patients were deemed compliant if they reported taking their prescribed medications and either discontinued or reduced their marijuana intake significantly. Cessation of marijuana use was not verified routinely with a drug screen.

### Data analysis

The data was analyzed using the Stata (StataCorp, College Station, TX). Categorical variables were summarized using proportions while continuous variables were summarized using means and standard deviation. The Fisher’s exact test or the chi-square test was used to perform between-group comparisons of categorical variables. The t-test was used for comparing continuous variables.

Univariate logistic regression was performed to identify variables associated with the dichotomous outcomes of interest, namely pediatric onset disease and response (complete / partial response vs. non-response) to therapy. Linear regression was used for continuous outcomes including number of hospitalizations, ED visits, duration of episodes and number of episodes. Variables that were significant in these regression models at p < 0.1 were selected for inclusion in the final multivariate model where p < 0.05 was indicative of independent statistical significance.

## Abbreviations

CVS, Cyclic Vomiting Syndrome; ED, Emergency Department; MCW, Medical College of Wisconsin; TCA, Tricyclic Antidepressant; OR, Odds Ratio; CI, Confidence Interval.

## Competing interests

The authors declare that they have no competing interests.

## Author contribution

NK Acquisition of the data Analysis and interpretation of the data Drafting of the manuscript Revisions and corrections. QB Collection of data Organization of the data Review of charts and follow-up. NR Acquisition of the data Review of literature Drafting of the tables/figures. JS Administrative, technical, or material support Organization of the data Review of literature. AA Statistical Analysis Critical review of manuscript. AS Acquisition of data Patient recruitment and follow-up. WJH Study concept and design Critical review of the manuscript. TV Study concept and design Drafting of manuscript Critical review of the manuscript Study overview.

## Pre-publication history

The pre-publication history for this paper can be accessed here:

http://www.biomedcentral.com/1471-230X/12/52/prepub
